# Malate targets pyruvate kinase M2 to promote colorectal cancer cell cycle arrest and tumor suppression

**DOI:** 10.1186/s43556-025-00326-y

**Published:** 2025-10-11

**Authors:** Kun Zhao, Fan Zhang, Qing Qin, Dapeng Zhang, Feng Yang, Yulan Huang, Renchao Deng, Huan Jing, Weidong Xiao, Hongming Miao, Rongchen Shi

**Affiliations:** 1 Department of Pathophysiology, College of High Altitude Military Medicine, Third Military Medical University (Army Medical University), No. 30 Gaotanyan Street, Shapingba, Chongqing, 400038 People’s Republic of China; 2 Key Laboratory of Extreme Environmental Medicine Ministry of Education of China, No. 30 Gaotanyan Street, Shapingba, Chongqing, 400038 People’s Republic of China; 3 Jinfeng Laboratory, No. 313, Jin Yue Road, High-tech Zone, Chongqing, 401329 People’s Republic of China; 4 Department of General Surgery, Xinqiao Hospital Third Military Medical University (Army Medical University), No. 183 Xinqiao Street, Shapingba, Chongqing, 400037 People’s Republic of China; 5 Frontier Medical Training Brigade Third Military Medical University (Army Medical University), Xinjiang, China

**Keywords:** Colorectal cancer (CRC), Malate, Pyruvate kinase M2 (PKM2), Cell division cycle 25 A (CDC25A), Cell cycle arrest

## Abstract

To survive nutrient stress caused by rapid proliferation and dysfunctional vasculature, tumor cells extensively reprogram their metabolic pathways, including the tricarboxylic acid (TCA) cycle representing a critical remodeling node. Functioning as a key TCA cycle intermediate, malate bridges fumarate and oxaloacetate, both of which are metabolites known to play significant roles in tumorigenesis. However, whether malate itself regulates tumor progression and the specific mechanism remain unclear. In this study, we demonstrate that oral administration of malate significantly inhibits the growth of colorectal cancer (CRC) xenografts in both nude mice and immunocompetent models, suggesting its antitumor effects are immunity-independent. Mechanistically, we found that malate acts as an allosteric regulator of pyruvate kinase M2 (PKM2), binding to it and initiating a cascade that promotes the ubiquitin-mediated proteasomal degradation of cell division cycle 25 A (CDC25A). This reduction in CDC25A enhances the inhibitory phosphorylation of CDK1 at Tyr15, leading to cell cycle arrest and suppression of proliferation. Clinical analyses further support these findings, showing decreased malate levels in human CRC tissues. Moreover, the expression of malate-metabolizing enzymes, MDH1 and FH, is significantly correlated with activity of the CDC25A/p-CDK1 signaling axis. Collectively, our results identify malate as a non-metabolic regulator of the cell cycle, operating through the PKM2-CDC25A-CDK1 pathway, and propose a novel therapeutic strategy targeting metabolic mediators of cell proliferation in cancer.

**Supplementary Information:**

The online version contains supplementary material available at 10.1186/s43556-025-00326-y.

## Introduction

Metabolic reprogramming represents a defining hallmark of cancer, facilitating oncogenesis and therapeutic resistance [1, 2]. This adaptive rewiring diverges fundamentally from normal cellular metabolism, characterized by enhanced glycolysis, dysregulated amino acid metabolism, and aberrant lipid biosynthesis, collectively working together to help tumors grow and make them adapt to their surroundings [3]. Beyond meeting biosynthetic demands, tumor-associated metabolic alterations generate signaling metabolites that regulate oncogenic pathways, thereby coordinating invasion, metastasis, and immune evasion [4]. Systematic characterization of these perturbations provides critical pathobiological insights and reveals novel therapeutic targets.

Tricarboxylic acid (TCA) cycle, as a central metabolic hub, orchestrates tumor progression through its multiple metabolites [5, 6]. Key intermediates, including α-ketoglutarate (α-KG) and succinate, could exert epigenetic regulation via histone/DNA demethylase modulation [7, 8], while fumarate and citrate could stabilize hypoxia-inducible factor 1α (HIF-1α) to enhance tumor cell survival [9, 10]. Notably, TCA metabolites demonstrate immunomodulatory capacities through microenvironmental reprogramming, establishing their dual role as metabolic substrates and signaling effectors [11]. These discoveries redefine TCA cycle components as critical nodes within the tumor regulatory network.

Existing researches have mainly focused on malate-metabolizing enzymes (MDH1/MDH2) in tumors [12–14], whereas the changes in malate levels and their functional significance in cancer progression remain unclear. Some evidences implicate malate could maintain redox homeostasis (NAD +/NADH balance), modulate reactive oxygen species (ROS) dynamics, and facilitate mitochondrial-cytoplasmic crosstalk through the malate-aspartate shuttle [15, 16], all of which may regulate tumor progression. Our prior investigation also revealed its regulatory role in macrophage polarization during inflammatory processes [17]. Thus, elucidating the functional role and mechanistic basis of malate in cancer progression remains crucial.

The precise coordination of cellular metabolism with the cell cycle is fundamental to proliferation. However, the molecular mechanisms linking cell metabolism to the core cell cycle remain incompletely defined. Cyclin-Dependent Kinases (CDKs) are the central to cell cycle progression, whose enzymatic activity is tightly regulated by specific phosphorylation states [18, 19]. The phosphatase cell division cycle 25 A (CDC25A) plays a crucial role in the phosphorylation process of CDKs, which can activate CDKs by dephosphorylation, thereby directly promoting the transitions from G1/S phase to G2/M phase [18]. Emerging evidence highlights the pyruvate kinase M2 isoform (PKM2), a pivotal glycolytic enzyme frequently upregulated in tumor cells, promotes transcription in G1 phase [20]. Research also has demonstrated that CDC25A interacts with PKM2 via direct or indirect mechanisms, influencing PKM2-mediated downstream effects [21, 22]. However, whether and how this reciprocal interaction modulates CDC25A-dependent cell cycle progression remains unclear. Further elucidation of these underlying mechanisms will provide an important theoretical basis for tumor treatment.

This study presents the first experimental demonstration of direct antitumor effect by malate in colorectal cancer (CRC) models. In vitro analyses demonstrated malate could induce tumor cell cycle arrest, while in vivo administration significantly inhibited subcutaneous xenograft growth. Mechanistically, we identified a novel PKM2-CDC25A-CDK1 axis, namely malate-binding PKM2, which triggered the ubiquitin–proteasome pathway of CDC25A and increased phosphorylation of CDK1, which could arrest tumor proliferation. These findings establish malate as a metabolic checkpoint regulator and expand therapeutic strategies targeting tumor metabolic plasticity.

## Results

### Malate inhibits subcutaneous CRC growth in mice

**Fig. 1:**
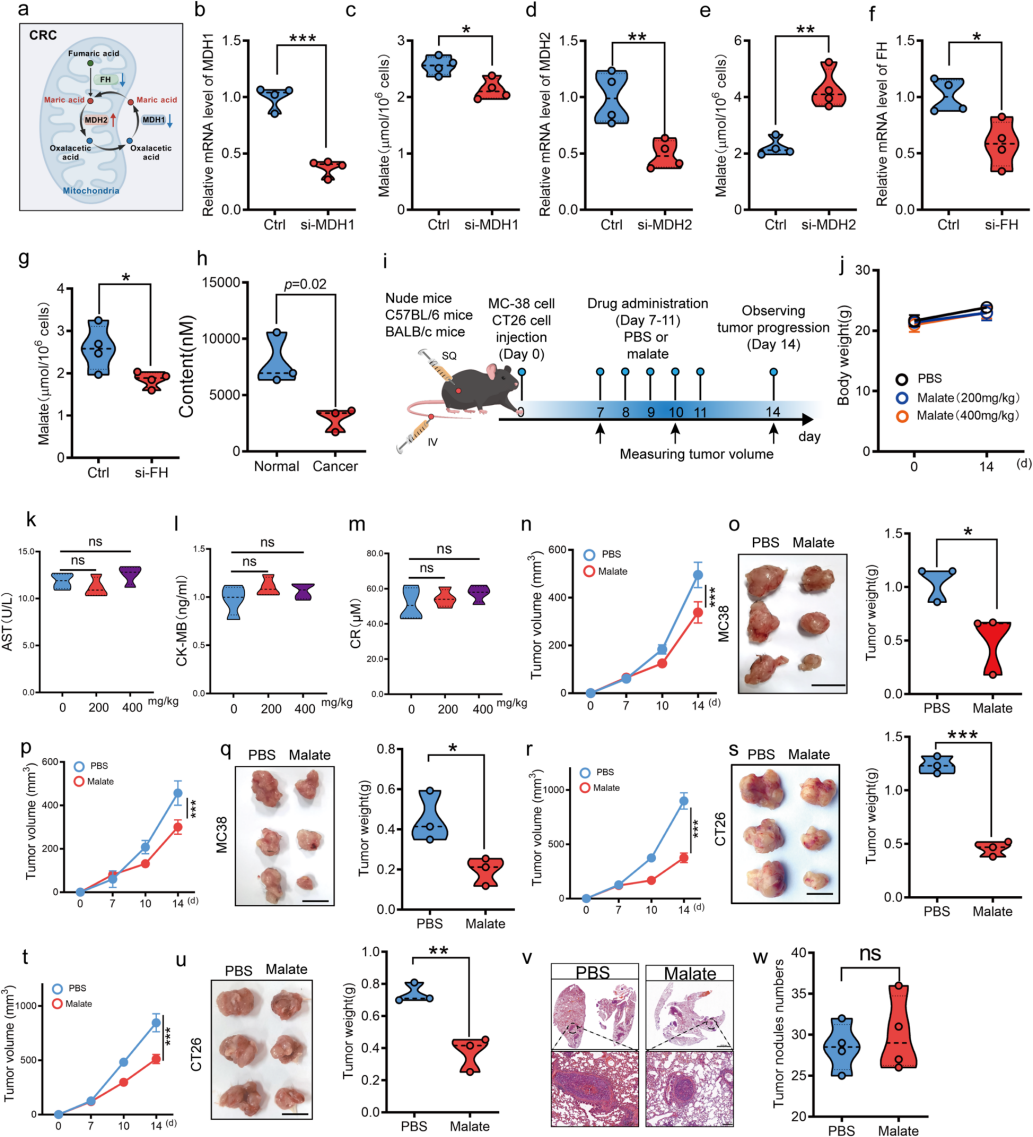
Oral administration of malate inhibits the growth of subcutaneous colorectal cancer in mice. **a**. Schematic diagram of malate metabolism within cells. In mitochondria, fumarate is converted to malate and then oxaloacetate, which is shuttled to the cytoplasm, reduced back to malate, and returned to mitochondria to sustain TCA cycle flux. **b**. The mRNA level of MDH1 in MC38 cells following siRNA-mediated silencing. (*n* = 4). **c**. The content of malic acid in Ctrl or si-MDH1 MC38 cells. (*n* = 4). **d**. The mRNA level of MDH2 in MC38 cells following siRNA-mediated silencing. (*n* = 4). **e**. The content of malic acid in Ctrl or si-MDH2 MC38 cells. (*n* = 4). **f**. The mRNA level of FH in MC38 cells following siRNA-mediated silencing. (*n* = 4). **g**. The content of malic acid in Ctrl or si-FH MC38 cells. (*n* = 4). **h**. The content of malate in cancer tissues and normal tissues of patients with CRC. (*n* = 3). **i**. Schematic diagram of malate treatment. 1 × 10^6^ MC38 or CT26 cells were injected into the male nude/C57/Babl/c mice at 6–8 weeks of age (subcutaneous or tail vein). One week later, oral administration of malate (200 mg/kg) was carried out daily for 5 days. On the fourteenth day, the mice were sacrificed to observe tumor progression. **j**. The body weight of mice. 1 × 10^6^ MC38 cells were subcutaneously injected into the groin of male C57 mice at 6–8 weeks of age. One week later, oral administration of malate (200 mg/kg or 400 mg/kg) was carried out daily for 5 days. On the fourteenth day, comparing the weight changes on the 1 st day and the 14th day. (*n* = 4). **k**-**m**. The serum aspartate transaminase (AST) (**k**), creatine Kinase(CK) (**l**), and creatinine (CR) (**m**) level in the mice of Fig. 1j. (*n* = 4). **n**–**o**. Malate treatment inhibits the progression of subcutaneous transplanted tumors in nude mice of MC38 cells. The tumor volume (**n**), the presentative images (**o**, left) and tumor weight (**o**, right). Experimental method is shown in Fig. 1j. (*n* = 3), scar bar:1 cm. **p**-**q**. Malate treatment inhibits the progression of subcutaneous transplanted tumors in C57 mice of MC38 cells. The tumor volume (**p**), the presentative images (**q**, left) and tumor weight (**q**, right). Experimental method is shown in Fig. 1j. (*n* = 3), scar bar:1 cm. **r**-**s**. Malate treatment inhibits the progression of subcutaneous transplanted tumors in nude mice of CT26 cells. The tumor volume (**r**), the presentative images (**s**, left) and tumor weight (**s**, right). Experimental method is shown in Fig. 1j. (*n* = 3), scar bar:1 cm. **t**-**u**. Malate treatment inhibits the progression of subcutaneous transplanted tumors in Babl/c mice of CT26 cells. The tumor volume (**t**), the presentative images (**u**, left) and tumor weight (**u**, right). Experimental method is shown in Fig. 1j. (*n* = 3), scar bar:1 cm. **v**-**w**. Malate treatment does not regulate the progression of lung metastasis of CRC in mice constructed with MC38. The presentative images (**v**) and tumor nodules numbers (**w**). Experimental method is shown in Fig. 1j. (*n* = 4), scar bar: 2000 μm (upper), Scar bar: 200 μm (under). All data represent the means ± s.e.ms. Except E–G were analyzed with Kaplan–Meier survival analysis, the rest were analyzed with Student's t test. (*P < 0.05, **P < 0.01, and n.s. not significant)

To investigate the metabolic role of malate in CRC progression, we conducted an integrated bioinformatics analysis of TCGA datasets, systematically profiling the expression patterns of important enzymes governing malate metabolism (synthesis/degradation) in CRC and the correlation with patient prognosis (Fig. 1a). We found that dysregulated malate anaplerotic enzymes (MDH1/MDH2/FH) may reduce cellular malate levels, correlating with poor prognosis (data not shown). To further confirm that the changes in the expression of MDH1/MDH2/FH would cause variations of the intracellular malic acid level, we silenced the expression of MDH1, MDH2, and FH in MC38 cells, and found that silencing MDH1/FH significantly decreased the malic acid level, while silencing MDH2 increased the malic acid level (Fig. 1b-g), suggesting metabolic rewiring toward malate depletion in CRC cells. Metabolome analysis of clinical samples also confirmed the content of malate in CRC was lower compared with adjacent tissues (Fig. 1h). Next, we constructed multiple mouse CRC models to explore the regulatory effect of malate on tumor progression (Fig. 1i). We found that different doses of malate administration did not cause significant toxic side effects in the mice (Fig. 1j-m). Importantly, malate significantly suppressed subcutaneous MC38 tumor growth in both nude mice (Fig. 1n-o) and immunocompetent C57BL/6 mice (Fig. 1p-q). Consistent results were found in CT26 cells (Fig. 1r-u). However, malate did not regulate the lung metastasis (Fig. 1v-w) of CRC in mice.

### Malate does not affect the tumor immune microenvironment of mice

**Fig. 2:**
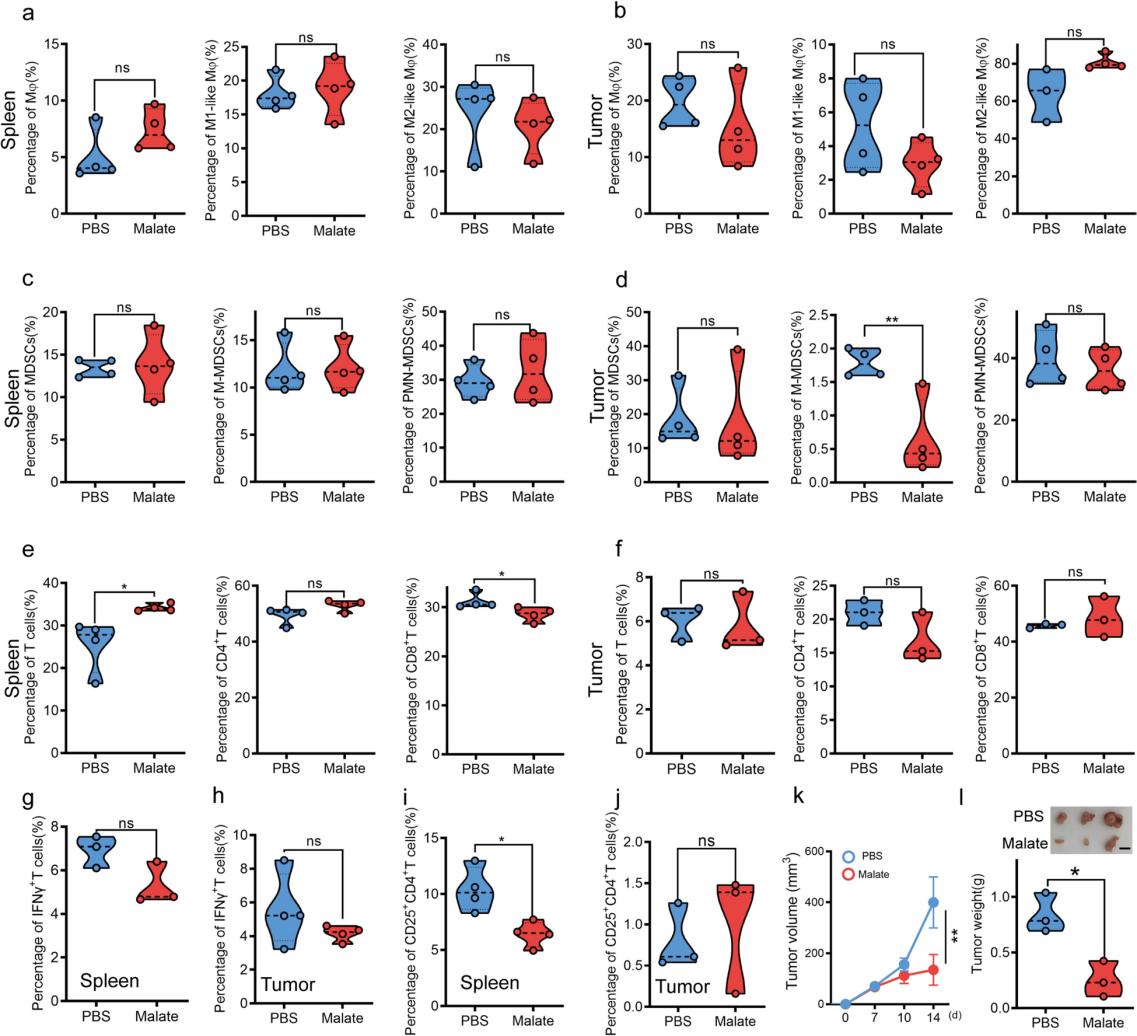
The antitumor effects of malate are immune-independent. **a**-**b**. Malate did not affect the proportion of total macrophages, M1-like (CD11c^+^) or M2-like (CD206^+^) in the spleen (**a**) or tumor (**b**) of mice. 1 × 10^6^ MC38 cells were subcutaneously injected into the groin of male nude mice at 6–8 weeks of age. One week later, oral administration of malate (200 mg/kg) was carried out daily for 5 days. On the fourteenth day, the mice were sacrificed to analyze the immune microenvironment of the spleen or tumor. (*n* = 4). **c**-**d**. Malate downregulated the proportion of M-MDSCs in mouse tumors without affecting the total number of MDSCs or PMN-MDSCs in the spleen (**c**) or tumors (**d**). (*n* = 4). **e**-**j**. Malate did not affect the proportion of T cells in the spleen (**e**, **g**, and **i**) or tumors (**f**, **h**, and **j**), as well as the number of their subtypes and the expression of IFN-γ. (*n* = 4 or 3). **k**-**l**. Malate inhibits the progression of subcutaneous transplanted tumors constructed by MC38 in myeloid cell clearance mice. The tumor volume (**k**), the presentative images (**l**, above) and tumor weight (**l**, below). (*n* = 3), scar bar:1 cm. All data represent the means ± s.e.ms. All data were analyzed with Student's t test. (*P < 0.05, **P < 0.01, and n.s. not significant)

Previous studies have demonstrated that malate inhibits M1-like macrophage activity and exhibits significant therapeutic efficacy in various inflammatory diseases [17]. To explore whether malate influenced tumor growth by modulating the tumor immune microenvironment (TIME), we analyzed immune cell populations in the spleen and tumor microenvironment (TME) of mice using flow cytometry (Fig. S1a). Our findings revealed that malate treatment did not alter the abundance or activation status of macrophages in either the spleen (Fig. 2a and Fig. S1b-c) or tumor (Fig. 2b and Fig. S1d-e). Similarly, while malate had no effect on the overall proportion of myeloid-derived suppressor cells (MDSCs) or polymorphonuclear MDSCs (PMN-MDSCs) in the spleen (Fig. 2c and Fig. S1f-g) or tumor (Fig. 2d and Fig. S1h-i), it significantly reduced the population of monocytic MDSCs (M-MDSCs) in tumor (Fig. 2d). Furthermore, malate did not affect the frequencies of total T cells, CD4⁺ T cells, CD8⁺ T cells, or IFN-γ⁺ T cells in either compartment (Fig. 2e-h and Fig. S1j-o). However, it selectively downregulated the proportion of CD25⁺ T cells in the spleen (Fig. 2i and Fig S1p) without influencing their prevalence in the tumor (Fig. 2j and Fig. S1q). To further investigate the potential role of monocytes in malate-mediated antitumor effects, we established the subcutaneous tumor model in monocyte-depleted mice. Intriguingly, malate could also inhibit tumor growth in these mice (Fig. 2k-l), suggesting that the antitumor effect is independent of monocytes. Collectively, these results indicated that malate may not regulate tumor immunity to inhibit tumor growth.

### Malate inhibits the proliferation of CRC cells by mediating cell cycle arrest

**Fig. 3:**
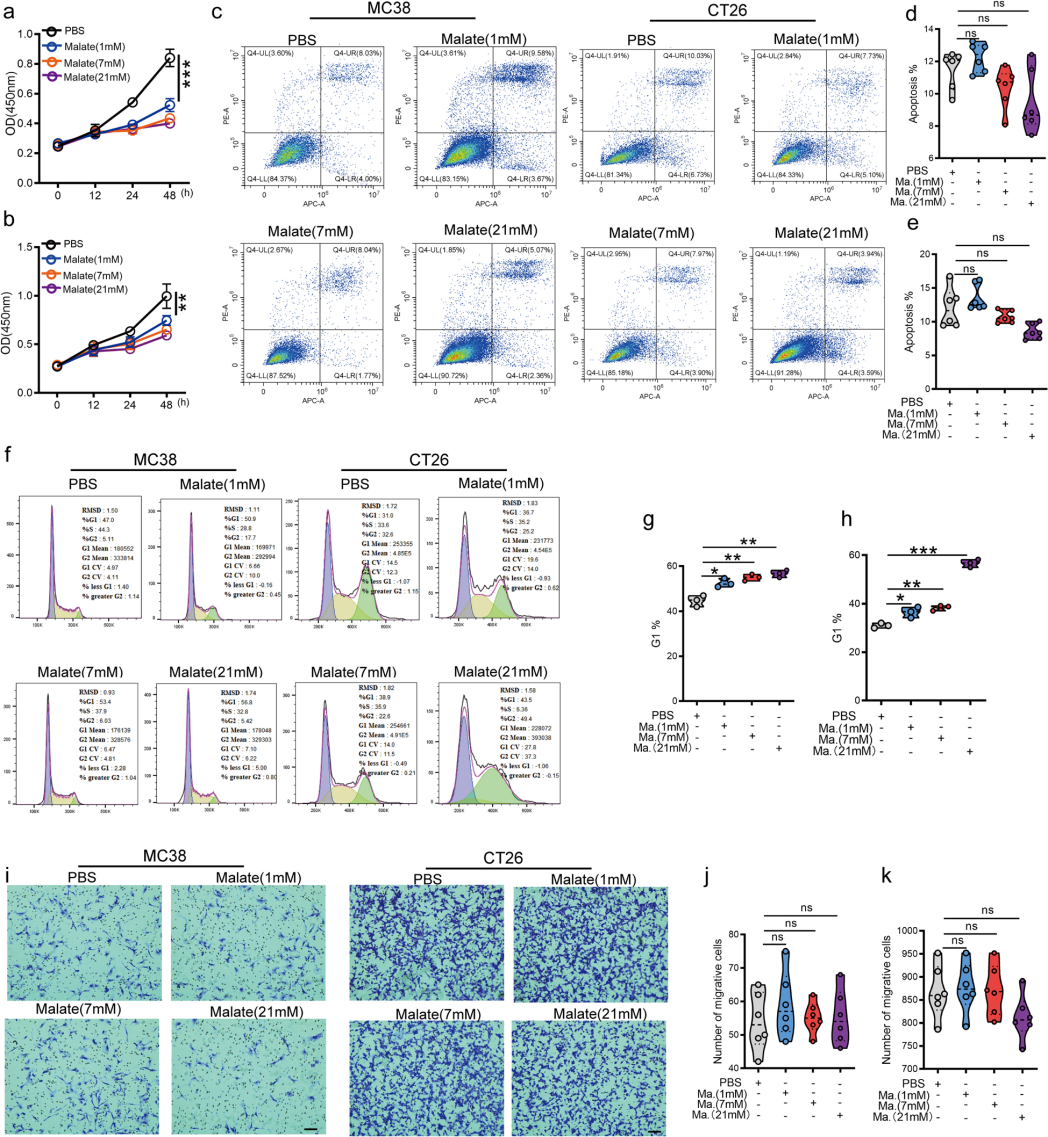
Malate mediates cell cycle arrest to inhibit the proliferation of CRC cells. **a**-**b**. Malate treatment significantly downregulated the proliferation activity of MC38 (**a**) or CT26 (**b**) cells. 2 × 10^3^ MC38 or CT26 cells were spread in 96-well plates, and the absorbance values at 450 nm were detected at 0, 12, 24, and 48 h, respectively. Before each test, discard the culture medium, add fresh culture medium containing Cell Counting Kit-8 (CCK-8), and continue to incubate for 60 min before testing. (*n* = 3). **c**-**e**. Malate treatment did not affect the apoptosis of MC38 or CT26 cells. The cell apoptosis was detected by flow cytometry after treating MC38 or CT26 cells with different concentrations of malate for 48 h. (*n* = 6). **f**–**h**. Malate treatment significantly promoted cell cycle arrest in MC38 and CT26 cells. The cell cycle was detected by flow cytometry after treating MC38 or CT26 cells with different concentrations of malate for 48 h. (*n* = 3). **i**-**k**. Malate treatment did not affect the migration of MC38 and CT26 cells. The cell migration was detected by crystal violet staining after treating MC38 or CT26 cells with different concentrations of malate for 48 h. (*n* = 6), Scar bar: 100 μm. All data represent the means ± s.e.ms. All data were analyzed with Student's t test. (*P < 0.05, **P < 0.01, ***P < 0.001, and n.s. not significant)

To elucidate the molecular mechanisms underlying the anti-tumor effect of malate, we investigated the impact of varying malate concentrations on the biological behaviors of CRC cells in vitro. Quantitative analysis demonstrated that malate treatment significantly suppressed the proliferative capacity of both MC38 (Fig. 3a) and CT26 (Fig. 3b) cells. However, flow cytometric analysis revealed no statistically significant changes in apoptosis following malate exposure (Fig. 3c-e). Notably, malate induced cell cycle arrest in a dose-dependent manner (Fig. 3f-h). Consistent with the observation that malate did not influence tumor metastasis in vivo, we also found that malate had no significant effect on tumor cell migratory activity (Fig. 3i-k). Collectively, these findings suggested that malate inhibited tumor growth primarily by promoting cell cycle arrest to reduce cell proliferative capacity.

### Malate promotes CRC cell cycle arrest through CDC25A/CDK1 signaling

**Fig. 4:**
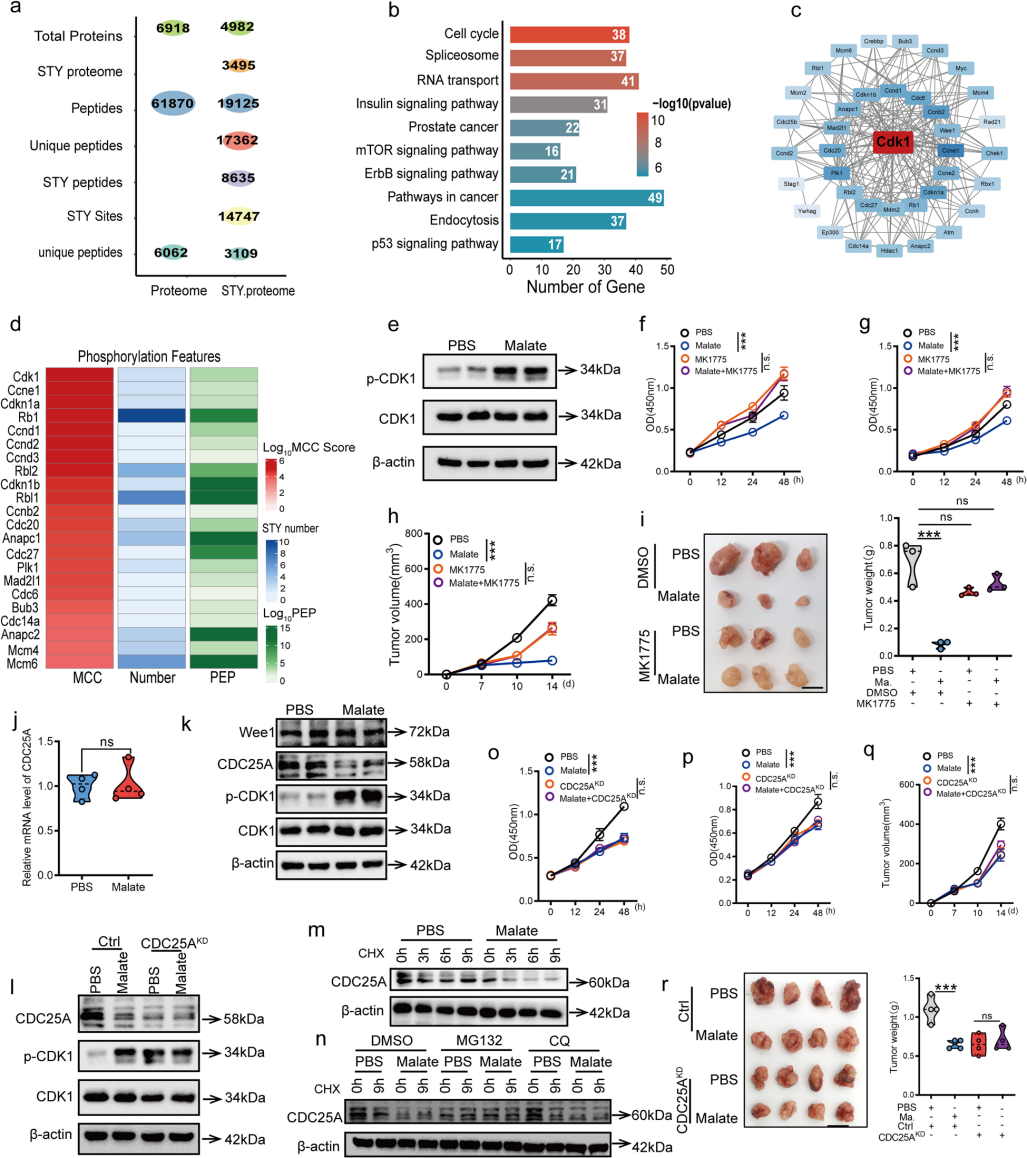
Malate mediates the inhibition of CRC cell proliferation and tumor growth by the CDC25A/CDK1 signaling pathway. **a**. Bubble chart shows the number of phosphorylation-modified peptides, phosphorylated sites, and proteins identified by 4D MS. **b**. Pathway analysis by the proteomic and phosphorylation proteomic analyses revealed cell cycle as the top protein-driven pathway. **c**. PPI network showing the proteins clustered in the cell cycle pathway and screening of hub genes using the MCC algorithm. **d**. The top 20 proteins were significantly associated with the pathways indicated in Fig. 4c. **e**. Malate treatment significantly promoted the phosphorylation levels of CDK1 in MC38 cells. After 48 h of malate treatment (7 mM), western blot was used to detect the protein levels of CDK1 and p-CDK1 in MC38 cells. **f**-**g**. The CDK phosphorylation inhibitor MK1775 eliminates the weakened effect of MC38 (**f**) and CT26 (**g**) cell proliferation mediated by malate treatment. **h**-**i**. The CDK phosphorylation inhibitor MK1775 eliminates the tumor growth delay effect mediated by malate treatment. 1 × 10^6^ MC38 cells were subcutaneously injected into the groin of male C57 mice at 6–8 weeks of age. MK1775 (30 mg/kg) was administered orally on the 3rd, 5th and 7th days. Then oral administration of malate (200 mg/kg) was carried out daily for 5 days from the seventh day. On the fourteenth day, the mice were sacrificed to observe tumor progression. The tumor volum (**h**), the presentative images (**i**, left) and tumor weight (**i**, right). (*n* = 3), scale bars, 1 cm. **j**. Malate did not affect the level of CDC25A mRNA. **k**. Malate treatment downregulated the protein level of CDC25A. After 48 h of malate treatment (7 mM), western blot was used to detect the protein levels of Wee1, CDC25A, CDK1 and p-CDK1 in MC38 cells. **l**. Malate cannot mediate the enhanced phosphorylation of CDK1 in MC38 cells with CDC25A knockdown (CDC25A^KD^). After 48 h of malate treatment (7 mM), western blot was used to detect the protein levels of CDC25A, CDK1, and p-CDK1 in Ctrl or CDC25A^KD^ MC38 cells. **m**. Malate promoted the degradation of CDC25A protein. Following 48-h malate treatment (7 mM) of MC38 cells, cycloheximide (CHX, 10 μg/mL) was administered to block protein synthesis. Cells were harvested at 0, 3, 6, 9, and 12 h post-CHX addition, and the protein level of CDC25A was detected by western blot. **n**. Malate-mediated degradation of CDC25A depends on the ubiquitin–proteasome pathway. **o**-**p**. Malate cannot inhibit the proliferation activity of CDC25A^KD^ MC38 (**o**) or CT26 (**p**) cells. **q**-**r**. Malate cannot inhibit the progression of subcutaneous transplanted tumors of CDC25A^KD^ MC38 cells. 1 × 10^6^ Ctrl or CDC25A^KD^ MC38 cells were subcutaneously injected into the groin of male C57 mice at 6–8 weeks of age. Then oral administration of malate (200 mg/kg) was carried out daily for 5 days from the seventh day. On the fourteenth day, the mice were sacrificed to observe tumor progression. The tumor volume (**q**), the presentative images (**r**, left) and tumor weight (**r**, right). (*n* = 4), scale bars, 1 cm. All data represent the means ± s.e.ms. All data were analyzed with Student's t test. (***P < 0.001, and n.s. not significant)

Given the critical role of protein phosphorylation in regulating CDKs and orchestrating cell cycle progression, we conducted a phosphoproteomic analysis to investigate the mechanism by which malate induced cell cycle arrest in CRC cells. Global proteomic profiling identified 6,918 proteins, while phosphoproteomic (STY) analysis quantified 4,982 proteins and 14,747 phosphorylation sites (Fig. 4a). KEGG enrichment analysis of overlapping proteins revealed that cell cycle-related phosphoproteins were the most significantly altered (Fig. 4b). Protein–protein interaction (PPI) network analysis constructed using the STRING database highlighted the CDK1 (Fig. 4c), a master regulator of the G2/M transition [18], as a key cell cycle regulator. A heatmap of the top 20 altered proteins ranked by maximal clique centrality (MCC) also indicated CDK1 (Fig. 4d) exhibited the most pronounced phosphorylation changes following malate treatment. Both phosphoproteomic and western blot analyses confirmed increased Y15 phosphorylation of CDK1 (Fig. 4e), suggesting their involvement in malate-induced cell cycle arrest. To validate the functional role of CDK1 phosphorylation, we treated MC38 and CT26 cells with malate combined with CDK phosphorylation inhibitors (MK1775), and found that malate-mediated reduction of cell proliferation activity disappeared after combined treatment with MK1775 (Fig. 4f-g). Notably, the anti-tumor effects of malate were also abolished (Fig. 4h-i), further supporting the dependence of malate-mediated growth suppression on CDKs phosphorylation.

Previous studies have established that CDK phosphorylation, particularly CDK1, is regulated by the opposing actions of WEE1 (an inhibitory kinase) and CDC25A (an activating phosphatase) [18, 19]. In our experiments, malate treatment significantly reduced CDC25A protein levels (Fig. 4k) in CRC cells without affecting its mRNA expression (Fig. 4j) and the protein level of Wee1 (Fig. 4k). Crucially, malate-induced CDK phosphorylation was abolished upon CDC25A knockdown (CDC25A^KD^) (Fig. 4l). Protein stability assays further demonstrated that malate accelerated CDC25A degradation (Fig. 4m). And the degradation of CDC25A mediated by malate is dependent on the proteasome (Fig. 4n). Consistent with this, both in vitro and in vivo experiments revealed that malate failed to suppress CRC cell proliferation (Fig. 4o-p) and subcutaneous tumor growth (Fig. 4q-r) in CDC25A^KD^ cells. In summary, our findings demonstrated that malate induced cell cycle arrest and inhibited tumor growth by modulating the CDC25A/CDK1 signaling axis.

### Malate binds PKM2 to regulate CRC cell proliferation and tumor progression

**Fig. 5:**
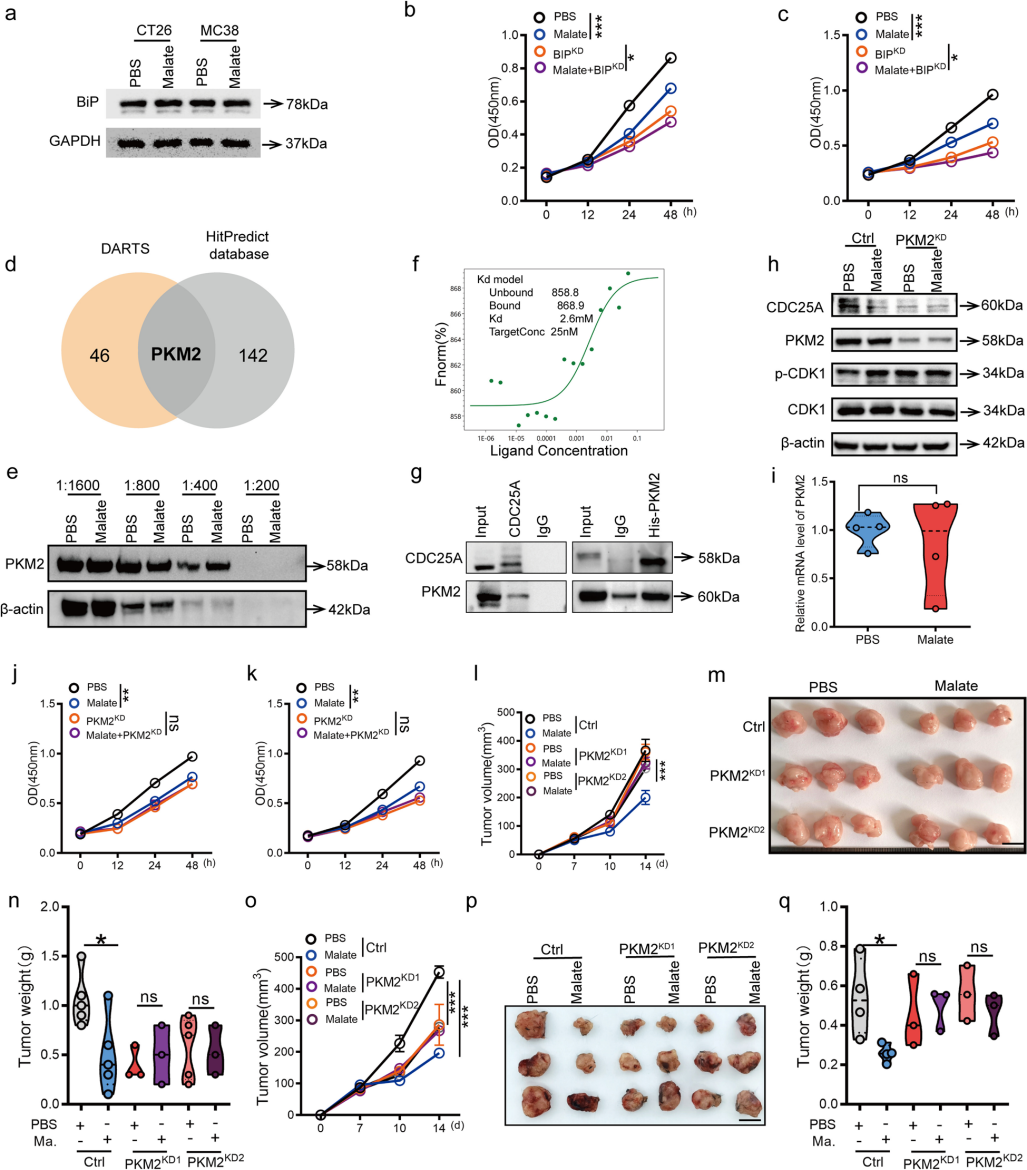
Malate-PKM2 binding suppresses CRC via CDC25A-CDK1 axis. **a**. Malate treatment did not affect the protein levels of BiP in MC38 and CT26 cells. After 48 h of malate treatment (7 mM), western blot was used to detect the protein levels of BiP in MC38 or CT26 cells. **b**-**c**. The knockdown of BiP cannot block the inhibitory effect of malate-mediated proliferation in MC38 (**b**) or CT26 (**c**) cells. **d**. Venn Diagram. The potential binding protein of malate and the interaction protein of CDC25A were used to screen out the key protein PKM2. **e**. Drug affinity responsive target stability (DARTS). After the MC38 cell protein was lysed and incubated with malate at 37 °C for 30 min, different concentrations of protease were added for treatment, and the reaction was immediately terminated on ice. The protein level of PKM2 was detected by the western blot experiment. **f**. Microscale thermophoresis (MST) was used to investigate the binding of malate with PKM2. **g**. The protein levels of PKM2 and CDC25A were detected by Co-IP assay. **h**. Malate cannot mediate the reduction of CDC25A protein and the enhanced phosphorylation of CDK1 in PKM2^KD^ tumor cells. After 48 h of malate treatment (7 mM), western blot was used to detect the protein levels of PKM2, CDC25A, CDK1 and p-CDK1 in Ctrl or PKM2^KD^ MC38 cells. **i**. Malate did not affect the mRNA level of PKM2. **j**-**k**. Malate cannot inhibit the proliferation activity of PKM2^KD^ MC38 (**j**) or CT26 (**k**) cells. (*n* = 5). **l**-**q**. Malate cannot inhibit the progression of subcutaneous transplanted tumors in PKM2^KD^ MC38 (**l**-**n**) or CT26 (**o**-**q**) cells. 1 × 10^6^ ctrl or PKM2.^KD^ MC38 or CT26 cells were subcutaneously injected into the groin of male C57/Babl/c mice at 6–8 weeks of age. Then oral administration of malate (200 mg/kg) was carried out daily for 5 days from the seventh day. On the fourteenth day, the mice were sacrificed to observe tumor progression. The tumor volume (**l** and **o**), the representative images (**m** and **p**) and tumor weight (**n** and **q**). (*n* = 5 or 3), scale bars, 1 cm. All data represent the means ± s.e.ms. All data were analyzed with Student's t test. (*P < 0.05, ***P < 0.001, and n.s. not significant)

To further investigate how malate regulates the CDC25A/CDK signaling pathway to influence CRC cell proliferation, we examined whether BiP, one of the most important molecular chaperone proteins in the endoplasmic reticulum and which has been reported to combine with maltae [17], mediated this process. Notably, malate exerted its anti-tumor effects independently of BiP regulation, as it neither changed BiP protein levels (Fig. 5a) nor lost efficacy upon BiP deletion (Fig. 5b-c). Instead, it still showed proliferation inhibition in BiP-knockdown cells (Fig. 5b-c), clearly pointing to other effector mechanisms. Subsequently, we employed drug affinity responsive target stability (DARTS) assays coupled with proteomic analysis to identify 46 potential malate-binding proteins (Fig. 5d). Cross-referencing these findings with the HitPredict database revealed 142 putative CDC25A-interacting proteins, among which PKM2 was the sole overlapping candidate (Fig. 5d). Further DARTS and microscale thermophoresis (MST) experiments confirmed the potential binding between malate and PKM2 (Fig. 5e-f). In addition, co-immunoprecipitation (Co-IP) assays verified the physical interaction between PKM2 and CDC25A (Fig. 5g). Notably, although malate did not modulate PKM2 mRNA or protein expression (Fig. 5h-i), its ability to downregulate CDC25A and enhance CDK1 phosphorylation was abolished upon PKM2 knockdown (PKM2^KD^) (Fig. 5h). These findings suggested that malate might promote CDC25A degradation and CDK1 phosphorylation by binding to PKM2. Critically, PKM2^KD^ also negated the inhibitory effects of malate on tumor cell proliferation (Fig. 5j-k) and subcutaneous tumor growth (Fig. 5l-q), underscoring the essential role of PKM2 in mediating the anti-tumor activity of malate.

### Malate allosterically binds PKM2 without altering its enzymatic activity or cellular glycolytic function

**Fig. 6:**
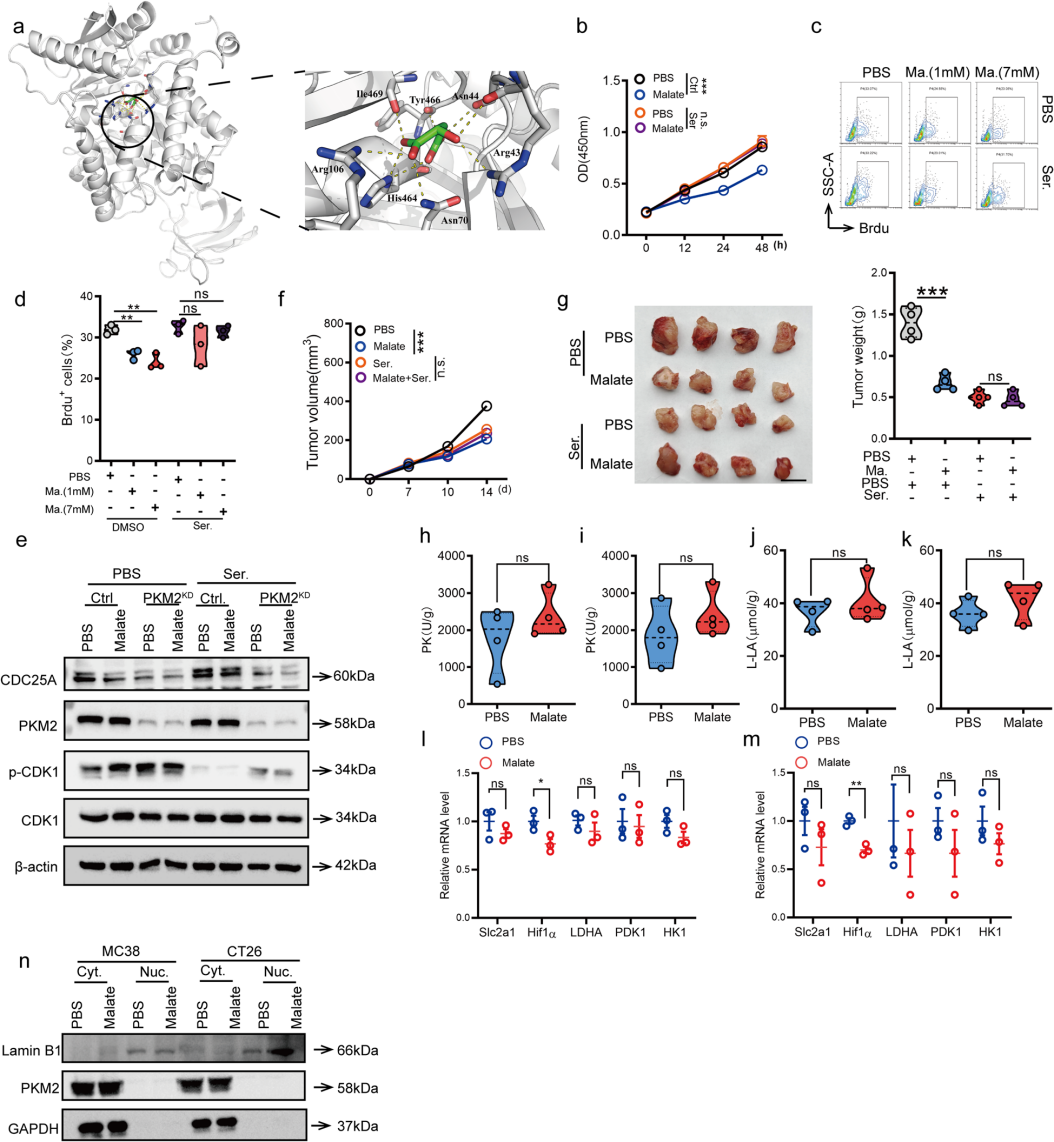
Malate allosterically binds PKM2 without perturbing its canonical activity. **a**. The molecule docking between malate and PKM2. **b**-**d**. Malate could not inhibit the proliferation activity of MC38 pretreated with Ser (200 μM, 6 h) by CCK8 (**b**) or BrdU (**c**-**d**) cell proliferation experiment. **e**. Malate cannot mediate the reduction of CDC25A protein and the enhanced phosphorylation of CDK1 after the pretreatment of Ser (200 μM, 6 h). **f**-**g**. Malate cannot inhibit the progression of subcutaneous transplanted tumors after the pretreatment of Ser. 1 × 10^6^ MC38 cells were subcutaneously injected into the groin of male C57 mice at 6–8 weeks of age. L-Serine (5 mg/kg) was administered intratumorally on the 3rd, 5th and 7th days. Then oral administration of malate (200 mg/kg) was carried out daily for 5 days from the seventh day. On the fourteenth day, the mice were sacrificed to observe tumor progression. The tumor volume (**f**), the presentative images (**g**, left) and tumor weight (**g**, right). (*n* = 4), scale bars, 1 cm. **h**-**i**. The PKM2 enzyme activity in tissues treated with malate. 1 × 10^6^ MC38 (**h**) or CT26 (**i**) cells were subcutaneously injected into the groin of male C57 mice at 6–8 weeks of age. Then oral administration of malate (200 mg/kg) was carried out daily for 5 days from the seventh day. On the fourteenth day, the mice were sacrificed to detect tumor tissue PKM2 enzyme activity. (*n* = 4). **j**. The lactic acid content in MC38 cells treated with malate (7 mM, 48 h). **k**. The lactic acid content in tumor tissues treated with malate. 1 × 10^6^ MC38 cells were subcutaneously injected into the groin of male C57 mice at 6–8 weeks of age. Then oral administration of malate (200 mg/kg) was carried out daily for 5 days from the seventh day. On the fourteenth day, the mice were sacrificed to detect tumor tissue lactic acid content. (*n* = 4). **l**-**m**. Malate treatment does not affect the expression of downstream genes regulated by PKM2. After 48 h of malate treatment (7 mM), the mRNA levels of Slc2a1, Hif1ɑ, LDHA, PDK1, and HK1 were detected in MC38 or CT26 cells. **n**. Malate treatment does not affect the nuclear translocation of PKM2. After 48 h of malate treatment, the protein levels of PKM2 in the cell nucleus and cytoplasm were detected separately. All data represent the means ± s.e.ms. All data were analyzed with Student's t test. (*P < 0.05, **P < 0.01, ***P < 0.001, and n.s. not significant)

To investigate the malate-PKM2 interaction, we predicted their binding configuration and identified key PKM2 residues (Arg43, Asn44, Asn70, Arg106, His464, Tyr466, and Ile469) that may form hydrogen bonds with malate (Fig. 6a). This binding mode is similar to the binding mode of serine in the PKM2 structure [23]. Molecular docking yielded a binding energy of −6.159 kcal·mol⁻^1^ for malate. To further validate malate's binding to PKM2 at the identified sites, we employed serine (Ser) to competitively inhibit malate binding in tumor cells. This pretreatment completely abolished the anti-proliferative effects of malate (Fig. 6b-d). Furthermore, the downregulation of CDC25A and the increase in CDK1 phosphorylation mediated by malate disappeared after the Ser pre-treatment (Fig. 6e). Consistent with these findings, co-administration of Ser also reversed malate-induced tumor growth delay (Fig. 6f-g).

PKM2 plays dual roles in cellular metabolism, functioning both as a critical glycolytic enzyme and as a transcriptional regulator that translocates to the nucleus to modulate gene expression [20]. To determine whether malate influenced these canonical PKM2 functions, we assessed PKM2 enzymatic activity, glycolytic flux (measured by lactate production), and nuclear translocation following malate treatment. We found that malate had no significant effect on PKM2 activity in tumor tissues (Fig. 6h-i). In line with these findings, malate treatment had no detectable effect on lactate levels in both cell and cancer tissue (Fig. 6j-k). Moreover, we found that malate neither influenced the expression of downstream target genes (Fig. 6l-m) nor affected PKM2 nuclear translocation (Fig. 6n). These results collectively demonstrated that malate exerted its PKM2-dependent antiproliferative activity principally by inducing cell cycle arrest via the CDC25A/p-CDK pathway, independent of PKM2 metabolic and transcriptional functions.

### Enzymes related to malate metabolism are correlated with the CDC25A-CDK1 signal pathway in CRC tissues

**Fig. 7:**
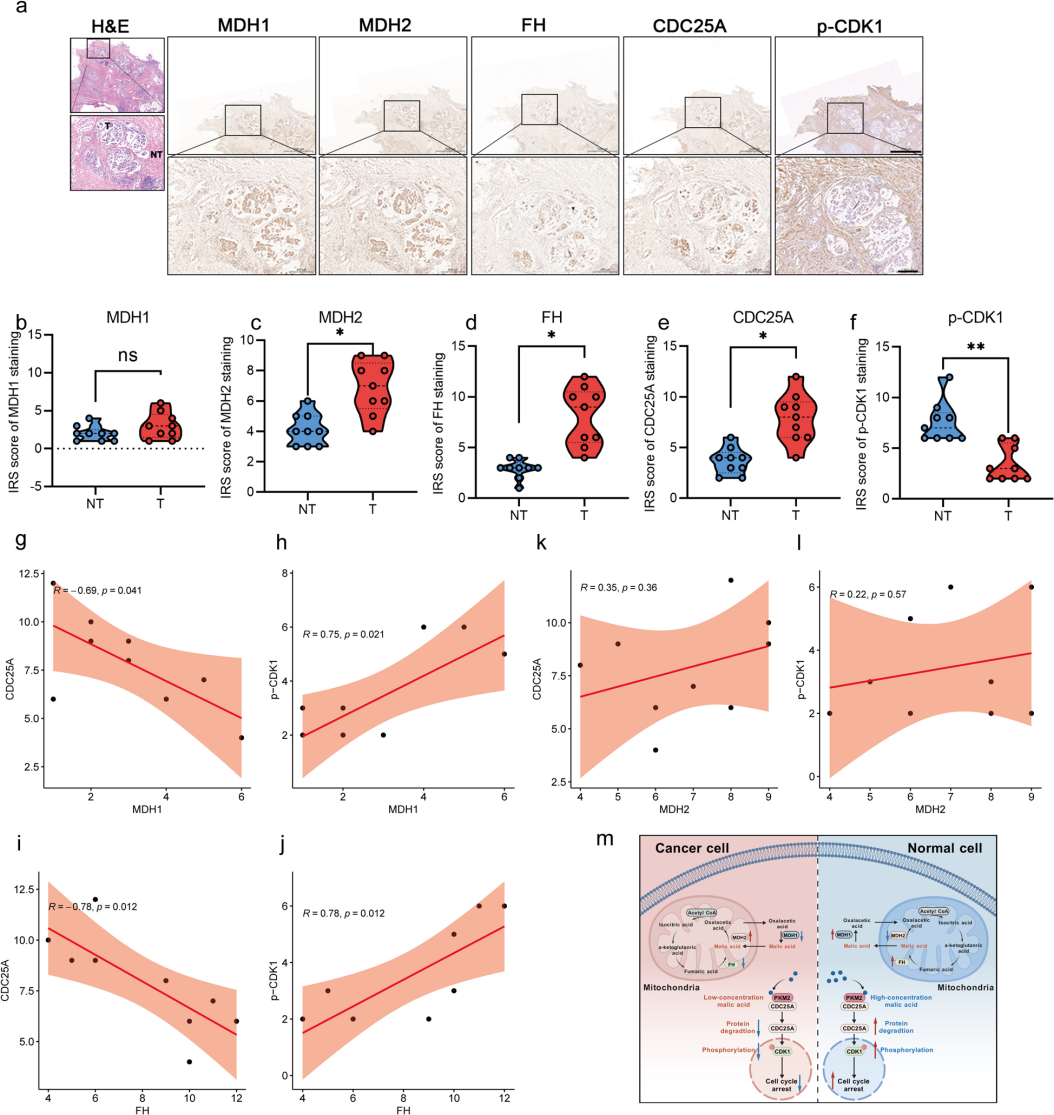
Clinical sample validation of the clinical relevance of the malate/PKM2/CDC25A/CDK1 pathway. **a**. The expressions of MDH1, MDH2, FH, CDC25A, and p-CDK1 in CRC tissues and corresponding adjacent tissues were analyzed by immunohistochemical staining (n = 9 pairs). Each sample was sectioned continuously and stained. The magnifications of the picture from top to bottom are 200 times and 400 times, respectively. Scar bar: 1000 μm (upper), Scar bar: 200 μm (under). **b**-**f**. The immunostained tissues were scored by IRS. **g**-**l**. The correlations between MDH1, MDH2, FH, and CDC25A, p-CDK1 based on the IPS scores. **m**. Graphical model. CRC tissues exhibit reduced malic acid levels compared to normal tissues, impairing its interaction with PKM2. This disruption upregulates CDC25A protein level, attenuates CDK1 phosphorylation, and abrogates cell cycle arrest, thereby driving tumor progression. Exogenous malate supplementation restores this regulatory axis, exerting anti-tumor effects. Created with BioGDP.com. All data represent the means ± s.e.ms. All data were analyzed with Student's t test. (*P < 0.05, **P < 0.01, and n.s. not significant)

To evaluate the clinical relevance of our findings, we performed immunohistochemical analysis on nine paired CRC specimens. While MDH1 expression remained comparable between tumor and adjacent normal tissues (Fig. 7a-b and Fig. S2), MDH2, FH, and CDC25A were significantly upregulated in malignant tissues (Fig. 7c-e), accompanied by markedly reduced CDK1 phosphorylation (Fig. 7f). Notably, MDH1 (Fig. 7g-h) and FH (Fig. 7i-j) exhibited a strong negative correlation with CDC25A expression but were positively correlated with CDK1 phosphorylation. In contrast, MDH2 showed no significant association with either CDC25A or phospho-CDK1 levels (Fig. 7k-l). These results established a compelling link between malate metabolism (particularly through MDH1/FH) and the dysregulation of CDC25A-CDK1 cell cycle axis in CRC.

## Discussion

This study established malate as a tumor-suppressive metabolite in CRC, selectively inhibiting subcutaneous tumor growth via cell cycle arrest. Mechanistically, malate depletion driven by dysregulation of MDH1 (down-regulation), MDH2 (overexpression), and FH (down-regulation) reduces the binding to PKM2 and degradation of CDC25A protein, thereby inhibiting the phosphorylation of CDK1 and ultimately reducing the tumor cell cycle arrest (Fig. 7m). These findings position malate as a novel metabolic regulator of the CDC25A/CDK axis, providing a theoretical basis for further exploring the clinical treatment based on the non-metabolic anti-tumor function of malate.

Notably, we identified a non-canonical PKM2 function, mediating CDC25A destabilization independent of its glycolytic or transcriptional roles. Structural analyses reveal malate allosterically occupies the PKM2 serine-binding pocket, selectively disrupting CDC25A stability without altering PKM2 enzymatic activity or glycolytic flux. Clinically, the inverse correlation between MDH1/FH expression and CDC25A levels in CRC specimens underscores malate metabolism as a prognostic biomarker for CDC25A-CDK1 pathway dysregulation.

In macrophages, malate binding to endoplasmic reticulum chaperone BiP stabilizes transcriptional repressor IRF2BP2, significantly reducing pro-inflammatory cytokine release (e.g., IL-1β), thereby attenuating chronic inflammation through metabolic-immune crosstalk [17]. Conversely, in tumor cells, malate binding to PKM2 triggers degradation of cell cycle regulator CDC25A, promoting CDK1 inhibitory phosphorylation (Tyr15) to suppress cell proliferation. This dual effect reveals the potential bridge between chronic inflammation and tumor transformation. On one hand, the continuous inhibition of inflammation by malate in macrophages may alleviate tissue damage, but in the long term, it may weaken the immune surveillance function and provide an escape opportunity for mutant cells. Concurrently, TME metabolic reprogramming (e.g., Warburg effect) disrupts malate homeostasis, simultaneously compromising anti-inflammatory regulation and activating oncogenic signaling. This mechanism suggests that targeting the malate pathway may intervene in the "inflammation-cancer transformation". For example, developing tissue-specific delivery strategies to increase malate concentration in the tumor area to activate the PKM2-CDC25A inhibitory axis, or supplementing malate analogues during the chronic inflammatory period to enhance the stability of IRF2BP2. Therefore, it is necessary to deeply explore how microenvironment factors (such as pH and metabolic enzyme spectrum) determine the "selection" of malate functions, and how tumor cells acquire malate resistance, providing new directions for metabolic intervention strategies.

Although we demonstrated that malate inhibits tumor growth in CRC via the PKM2/CDC25A/CDK1 axis, there are still some limitations that need to be further explored. For instance, the upstream drivers of dysregulated malate-metabolizing enzymes remain elusive. Whether genetic alterations (e.g., mutations, copy number variations), epigenetic modifications, or transcriptional regulation induced by tumor govern these expressions is undetermined. Meanwhile, the relative contribution of each enzyme to malate content in tumors is not quantified. Further investigation into which of the enzymes MDH1, MDH2 or FH plays a dominant role in regulating the content of malate is of great significance for the formulation of targeted treatment. In addition, whether there are potential interfering effects between these enzymes and other metabolic/signaling pathways (such as the adaptive response triggered by HIF-1ɑ, and the energy sensing of AMPK), and whether such interference affects the content of malate or the interaction between PKM2 and CDC25A also need to be further addressed. Finally, given that the reduction of FH will also lead to an increase of fumaric acid, which is a metabolite that has been reported to have significant carcinogenic effects, the tumor-suppressing effect of malate mediated by FH can also be interfered by fumaric acid, which requires special attention. Addressing these deficiencies is of crucial importance for the formulation of targeted malate tumor treatment strategies and clinical application.

In conclusion, malate plays the role of a metabolic checkpoint in CRC through the PKM2-CDC25A-CDK1 signaling axis, suggesting a therapeutic strategy that utilizes its cell cycle-specific effects to overcome PKM2-related chemotherapy resistance. Future research should explore the determinants of the anti-tumor efficacy of malate and optimize the treatment methods for metastatic lesions.

## Material and methods

### Cell culture

This study used two murine colon adenocarcinoma cell lines (CT26 and MC38) maintained in our laboratory. All cell lines underwent short tandem repeat (STR) profiling for authentication and demonstrated no harmful mycoplasma contamination through PCR-based testing. Cells were cultured in Dulbecco's Modified Eagle Medium (DMEM; #C3113-0500, VivaCell) supplemented with 10% fetal bovine serum (FBS; #C04001-500, VivaCell) and 1% penicillin–streptomycin solution (#C0222, Beyotime), maintained at 37 °C in a humidified 5% CO_2_ incubator.

### Mice

Specific pathogen-free (SPF) male C57BL/6 J mice (6–8 weeks old) and BALB/c mice (6–8 weeks old) were obtained from Hunan SJA Laboratory Animal Co., Ltd. Additionally, BALB/c nude mice (4–6 weeks old) were procured from Changzhou Cavens Model Animal Co., Ltd. CD11b-DTR transgenic mice (MacrophageKO) were used for myeloid depletion. DT administration (5 mg/kg IP) ablated CD11b⁺ cells via DTR expression at the CD11b locus. The Animal Care and Use Committee of the Army Medical University (AMU) has reviewed and approved the protocol for the use of animals in this study. All mice were handled and cared for in strict accordance with the ethical guidelines set forth by AMU's Animal Care and Use Committee.

### Mouse models of subcutaneous tumor (S.C)

Tumor cells were digested into single-cell suspension and resuspended with pre-cold PBS. Male mice received subcutaneous injections of 1 × 10⁶ tumor cells in 100 μL PBS using a 27-gauge needle. Tumor-bearing mice were humanely euthanized on day 14 post-injection. Excised tumors were immediately weighed and measured with digital calipers, with tumor volume calculated using the formula: Volume = (length × width^2^)/2.

### Mouse models of lung metastasis of CRC

Tumor cells were digested into single-cell suspension and resuspended with cold PBS. Male mice were injected via the tail vein using 27-gauge needle (1 × 10⁶ tumor cells in 100 μL suspension/mouse). The mice were sacrificed, and the tumor lung metastasis was measured after 2 weeks.

### CCK8 proliferation assay

Cell proliferation was quantified using the Cell Counting Kit-8 assay (#BS350B, Biosharp). CT26 and MC38 cells were plated in 96-well culture plates at a density of 2 × 10^3^ cells/well. Following incubation periods of 0, 12, 24, and 48 h, 10% CCK-8 solution was added to each well, and the plates were further incubated for 1 h at 37 °C under 5% CO₂. Absorbance at 450 nm was measured using a microplate spectrophotometer. Background absorbance values (blank wells containing medium and CCK-8 without cells) were subtracted from all experimental measurements before data analysis.

### AnnexinV-PI apoptosis assay

Plant 5 × 10^5^ tumor cells in a six-well plate cultured at 37 °C and 5% CO2 for 24 h with 2 mL of culture medium per well. Digestion with trypsin to obtain treated and untreated cells (1 × 10⁶/ml) and processed according to the instructions of the Annexin V/PI staining kit (BioLegend, USA), followed by analysis using flow cytometry (BD FACSVerse C6, USA) and FlowJo 10.8.1 software.

### Cell cycle assay

Following trypsinization, cells were fixed in ice-cold 70% ethanol at 4℃ overnight. After fixation, cells were washed twice with PBS and treated with RNase A (50 μg/mL) at 37 °C for 30 min to remove RNA interference. Subsequently, cells were stained with PI (50 μg/mL) in the dark at room temperature for 15 min. DNA content was measured using flow cytometer (BD FACSymphony A1, USA), and cell cycle phase distribution (G0/G1, S, and G2/M) was quantified using FlowJo software (Version 10.8.1; BD Life Sciences).

### Transwell assay

Cell migration was evaluated using 8-μm pore Transwell chambers (#353,097, Falcon). Cells suspended in 500μL FBS-free medium (1 × 10^5^/ml) were planted in the upper chamber, while the lower chamber contained 700μL of complete medium with 10% FBS as a chemoattractant. Following 24 h incubation at 37 °C with 5% CO₂, non-migrating cells were gently removed from the upper membrane surface using a cotton swab. Five random microscopic fields per membrane were counted after the migrated cells on the lower surface were fixed with 4% paraformaldehyde and stained with 0.1% crystal violet.

### Western blot

Cell proteins were extracted using RIPA lysis buffer (#P0013K, Beyotime) containing protease inhibitors and phosphatase inhibitors (#P1045, Beyotime) and quantified using the BCA kit (#P0012S, Beyotime). Each protein sample was separated by SDS-PAGE gel electrophoresis, and the isolated protein was transferred to a polyvinylidene fluoride membrane. The membrane was blocked with 5% skim milk powder and incubated with primary antibody for 10 h at 4 °C. Rinse the membrane with PBS containing 0.1% Tween 20 (#ST825-100 ml, Beyotime) for 5 min each time. Secondary antibodies coupled to horseradish peroxidase were incubated at 37 °C for two hours. Wash the membrane thoroughly 5 times with PBS containing 0.1% Tween-20. After stimulating the signal with an enhanced chemiluminescent substrate (PerkinElmer) for 1 min, the detection was performed using the Bio-Rad ChemiDoc MP system. Primary antibodies used in this experiment include P-CDK1, 2, 3, 5 (#ab133463-40ul, Abcam), β-actin (#66,009–1-IG-100UL, Proteintech), CDC25A (#55,031-AP-100ul, Proteintech), PKM2 (#60,268–1-1 g, Proteintech), BIP (#11,587–1-AP, Proteintech), Lamin B1 (#12,987–1-AP, Proteintech), WEE1 (#29,474–1-AP, Proteintech), CDK1 (#A11420, Abclonal), and p-CDK1 (#AP1350, Abclonal).

### Co-IP assay

Cells were collected and lysed in IP lysis buffer (#P0013J, Beyotime) in the presence of protease inhibitors and phosphatase inhibitors (#P1045, Beyotime) for 20 min and centrifuged at 4 °C for 10 min at 12,000 g. The grouped lysates were incubated with the indicated primary antibody or IgG (negative control, #A7016, Beyotime) overnight at 4℃ with constant rotation before incubating the cell lysates with protein A + G beads (#P2105, Beyotime) for an additional 2 h at room temperature. Cell lysates were washed 3 times with TBST, boiled at 95 °C for 10 min, and analyzed by western blot.

### Pyruvate Kinase (PK) activity assay

The treated cells and tissues were collected and measured using a pyruvate kinase (PK) activity assay kit according to the manufacturer's protocol (Solarbio, Beijing, China). The absorbance value of 340 nm was measured by a microplate reader to determine pyruvate kinase (PK) activity.

### Lactic Acid (LA) Content Assay

The treated cells and tissues were collected and measured with a lactic acid (LA) assay kit according to the manufacturer's protocol (#BC2335, Solarbio, China). The absorbance was measured at 570 nm using a microplate reader to determine the lactic acid level.

### Real-time PCR

Total RNA was extracted using a kit provided by ES Science (#RN001, ES Science). Thermo SCIENCE (AB-1771) was used for concentration determination, and 1000 ng of RNA was reverse transcribed into cDNA according to PrimerScriptRT kit (#RR036A, TaKaRa). Quantitative polymerase chain reaction with a total capacity of 20 μL was performed on a real-time fluorescence quantitative PCR system (Bio-Rad CFX Connect™ Optics Module) according to the TBGreen Premix Kit (#RR820A, TaKaRa). The expression level was normalized to the expression level of GAPDH, and relative expression was calculated.

### BrdU proliferation assay

To evaluate cell proliferation, tumor cells (3 × 10^5^ cells per well) were seeded into six-well plates containing 2 mL of culture medium and incubated for 24 h. After this initial incubation, BrdU reagent was added to the culture, followed by an additional incubation period of six hours. Cell proliferation was then quantified using a BrdU cell proliferation kit (#370,706, BioLegend) in accordance with the manufacturer's protocol.

### Drug Affinity Responsive Target Stability(DARTS)

Total protein extracted from MC38 cells was quantified, and 20 μg aliquots (20 μL) were loaded per well in an 8-row plate. After adding 1 μL PBS or malate (100 μM, 1 mM, or 7 mM), samples were incubated at 37 °C for 30 min. Protease (2 μL at indicated dilutions) was added followed by 10-min room temperature incubation. Reactions were quenched on ice. Samples were denatured with 5 × loading buffer (95 °C, 10 min) and subjected to western blotting for the protein level of PKM2.

### MicroScale Thermophoresis (MST)

Recombinant mice PKM2 protein was labeled with NT-647 NHS ester per manufacturer’s protocol. Malate (Sigma-Aldrich) was serially diluted (0.01–100 μM) in MST buffer (50 mM Tris–HCl pH 7.4, 150 mM NaCl, 0.05% Tween-20). Labeled PKM2 was mixed with diluted malate, and incubated 10 min at 25 °C. MST measurements were performed using Monolith NT.115 at 25 °C, 40% LED power, 60% MST power. Each concentration was tested in triplicate. Data were analyzed with MO.Control software; dissociation constant (Kd) was calculated via nonlinear regression to determine binding affinity.

### Malic acid Content Assay

The treated cells and flow-sorted cells were collected and measured with a Malic Acid Content Assay kit according to the manufacturer's protocol (#BC5945, Solarbio, China). The absorbance was measured at 505 nm using a microplate reader to determine the malic acid level.

### Glutamic-oxalacetic Transaminase (GOT) Assay

The mice serum was collected and measured with a Micro Glutamic-oxalacetic Transaminase (GOT) Assay Kit according to the manufacturer's protocol (#BC1565, Solarbio, China). The absorbance was measured at 450 nm using a microplate reader to determine the malic acid level.

### Creatine Kinase(CK) Activity Assay

The mice serum was collected and measured with a Creatine Kinase(CK) Activity Assay Kit according to the manufacturer's protocol (#BC1145, Solarbio, China). The absorbance was measured at 340 nm using a microplate reader to determine the malic acid level.

### Creatinine (CR) Content Assay

The mice serum was collected and measured with a Creatinine (CR) Content Assay Kit according to the manufacturer's protocol (#BC4910, Solarbio, China). The absorbance was measured at 505 nm using a microplate reader to determine the malic acid level.

### Flow cytometry

Mouse spleens were mechanically ground and filtered through 70 μm cell filter screen. Centrifuged cells underwent RBC lysis to generate single-cell suspensions. Tumor tissues were minced (1 mm^3^ fragments) and digested for 1 h in collagenase II (1 mg/mL), hyaluronidase (0.1 mg/mL), and DNase (0.01 mg/mL). Then cells were filtered, centrifuged, and lysed to obtain single-cell suspensions. For T-cell analysis, cells underwent 4–6 h activation (Biolegend, #423,303) prior to staining. Fc receptors were blocked before surface marker staining. Intracellular markers required fixation/permeabilization (Thermo Fisher Foxp3 Buffer #00–5523-00) prior to antibody staining. Flow cytometry antibodies include CD45 (Biolegend, #157,208/147716), F4/80 (Biolegend, #157,316), CD11c (Biolegend, #117,308), CD206 (Biolegend, #141,707), CD11b (Biolegend, #101,206), Ly6C (Biolegend, #128,014), Ly6G (Biolegend, #127,624), CD3 (Biolegend, #100,206), CD4 (Biolegend, #100,526), CD8 (Biolegend, #100,734), and IFNγ(Biolegend, #505,810).

### Statistical analysis

Analyzed data with GraphPad Prism (GraphPad Software, Inc.). Data was expressed as mean ± s.e.m and analyzed with Student’s test or Gehan-Breslow-Wilcoxon test. For each parameter of all data presented, n.s. means not significant, *P < 0.05, **P < 0.01, and ***P < 0.001.

## Supplementary Information

Supplementary Material 1.

Supplementary Material 2.

## Data Availability

All data are available from the corresponding authors upon request.
