# Supplementary material for: Malate targets pyruvate kinase M2 to promote colorectal cancer cell cycle arrest and tumor suppression
Source: Mol Biomed. 2025 Oct 11;6:79. doi: 10.1186/s43556-025-00326-y (PMC12515184; doi:10.1186/s43556-025-00326-y)
Supplement: Supplementary file 1 — Supplementary Material 1. [file 43556_2025_326_MOESM1_ESM.docx]

**Malate targets pyruvate kinase M2 to promote colorectal cancer cell cycle arrest and tumor suppression**

Kun Zhao^1,2,#^, Fan Zhang^1,2,#^, Qing Qin^1,2,#^, Dapeng Zhang^1,2,3^, Feng Yang^4^, Yulan Huang^1,2^, Renchao Deng^1,2^, Huan Jing^1,2^, Weidong Xiao^4,*^, Hongming Miao^1,2,3*^, Rongchen Shi^1,2,5,*^

1Department of Pathophysiology, College of High Altitude Military Medicine, Third Military Medical University (Army Medical University), Chongqing 400038, China.

2Key Laboratory of Extreme Environmental Medicine Ministry of Education of China Chongqing China.

3Jinfeng Laboratory, Chongqing 401329, China.

4Department of General Surgery Xinqiao Hospital Third Military Medical University (Army Medical University) Chongqing China.

5Frontier Medical Training Brigade Third Military Medical University (Army Medical University) Xinjiang China.

#Theses authors contributed equally to this work.

Correspondence to:

Rongchen Shi, No. 30 Gaotanyan Street, Shapingba, Chongqing 400038, People’s Republic of China, rongchenshitmmu@sina.com, Tel: 86-18875031656.

Hongming Miao, No. 30 Gaotanyan Street, Shapingba, Chongqing 400038, People’s Republic of China, hongmingmiao@sina.com, Tel: 86-13678484686.

Weidong Xiao, No. 183 Xinqiao Street, Shapingba, Chongqing 400038, People’s Republic of China, xiaoweidong@tmmu.edu.cn.

**Supplementary Information**


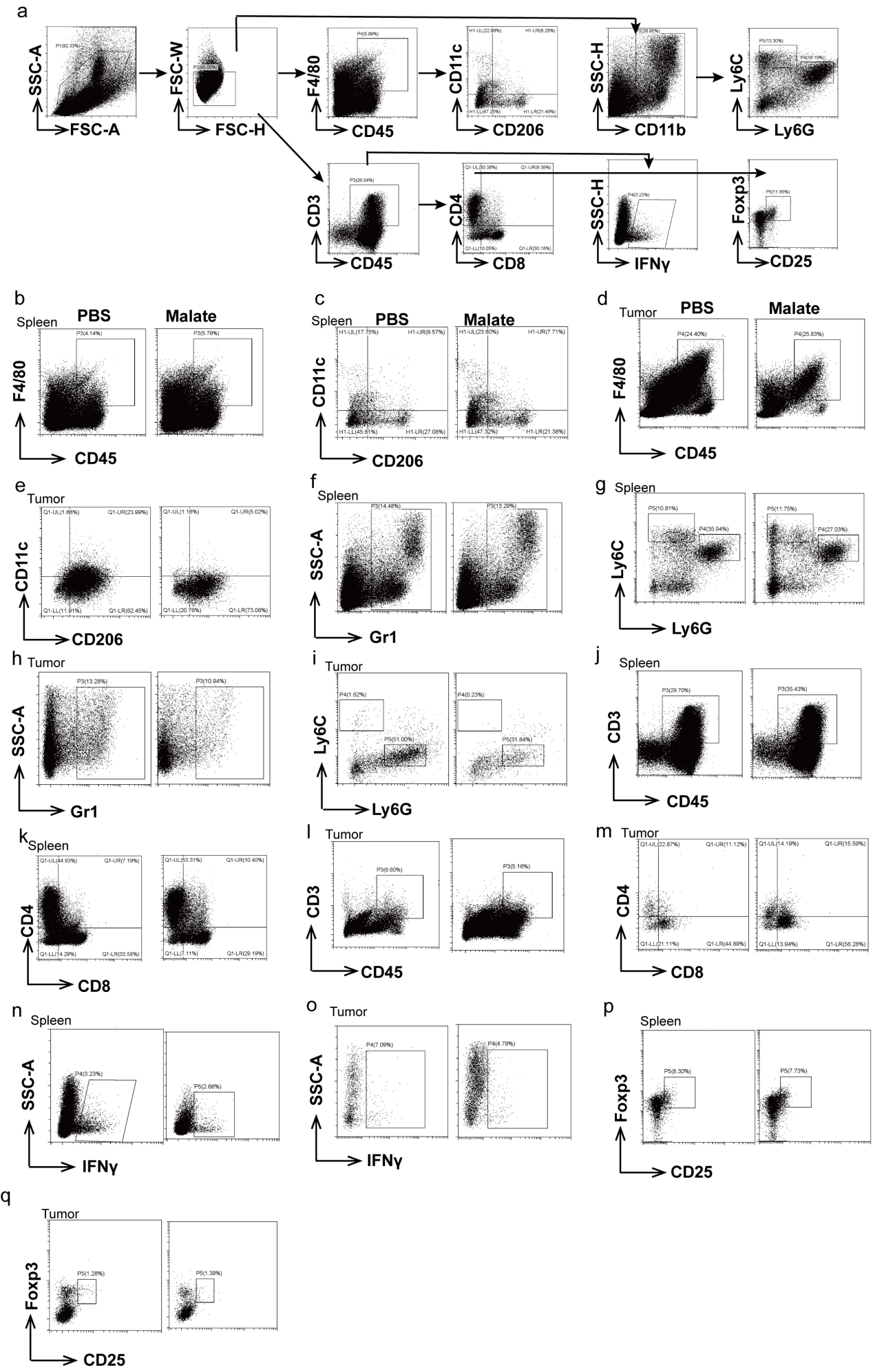


Fig S1. Malate treatment does not affect the immune system in mice.

a. Schematic diagram of immune cell analysis strategy. Macrophage is defined as CD45^+^F4/80^+^, M1-like macrophage is defined as CD45^+^F4/80^+^CD11c^+^CD206^-^, M2-like macrophage is defined as CD45^+^F4/80^+^CD11c^-^CD206^+^, PMN-MDSCs are defined as CD45^+^CD11b^+^Ly6C^low^Ly6G^+^, PMN-MDSCs are defined as CD45^+^CD11b^+^Ly6C^hi^Ly6G^-^, T cells are defined as CD45^+^CD3^+^, CD4^+^T cells are defined as CD45^+^CD3^+^CD4^+^CD8^-^, CD8^+^T cells are defined as CD45^+^CD3^+^CD4^-^CD8^+^, IFNγ^+^T cells are defined as CD45^+^CD3^+^IFNγ^+^, and Treg cells are defined as CD45^+^CD3^+^CD4^+^CD25^+^Foxp3^+^.

b-e. The proportion of total macrophages, M1-like or M2-like in the spleen or tumor of mice. 1×10^6^ MC38 cells were subcutaneously injected into the groin of male nude mice at 6-8 weeks of age. One week later, oral administration of malate (200 mg/kg) was carried out daily for 5 days. On the fourteenth day, the mice were sacrificed to analyze the immune microenvironment of the spleen or tumor. (n=3)

f-i. The percentage of MDSCs, MDSCs and PMN-MDSCs in the spleen or tumors. (n=3)

j-q. The proportion of T cells, CD4^+^ T cells, CD8^+^ T cells, IFNγ^+^ T cells, and Tregs in the spleen or tumors. (n=3)

Representative pictures were displayed.


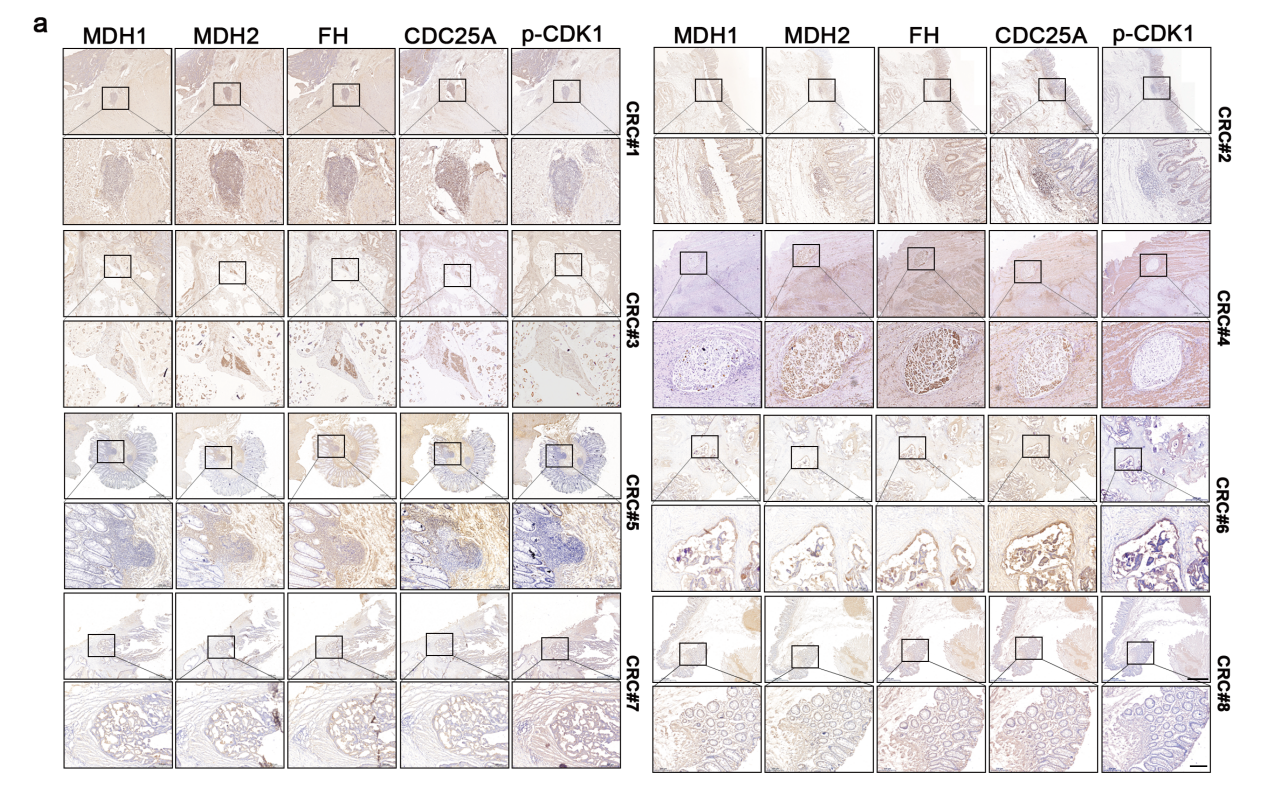


Fig S2. The expression of MDH1/FH is associated with the CDC25A/p-CDK1 signaling pathway.

a. The expressions of MDH1, MDH2, FH, CDC25A, and p-CDK1 in CRC tissues and corresponding adjacent tissues were analyzed by immunohistochemical staining (n = 9 pairs). Each sample was sectioned continuously and stained. The magnifications of the picture from top to bottom are 200 times and 400 times, respectively. Scar bar: 2000 μm (upper), Scar bar: 200 μm (under).
